# Intelligent Shape-Morphing Micromachines

**DOI:** 10.34133/2021/9806463

**Published:** 2021-05-12

**Authors:** Qianying Chen, Pengyu Lv, Jianyong Huang, Tian-Yun Huang, Huiling Duan

**Affiliations:** ^1^State Key Laboratory for Turbulence and Complex Systems, Department of Mechanics and Engineering Science, BIC-ESAT, College of Engineering, Peking University, Beijing 100871, China; ^2^CAPT, HEDPS, Peking University, Beijing 100871, China

## Abstract

Intelligent machines are capable of switching shape configurations to adapt to changes in dynamic environments and thus have offered the potentials in many applications such as precision medicine, lab on a chip, and bioengineering. Even though the developments of smart materials and advanced micro/nanomanufacturing are flouring, how to achieve intelligent shape-morphing machines at micro/nanoscales is still significantly challenging due to the lack of design methods and strategies especially for small-scale shape transformations. This review is aimed at summarizing the principles and methods for the construction of intelligent shape-morphing micromachines by introducing the dimensions, modes, realization methods, and applications of shape-morphing micromachines. Meanwhile, this review highlights the advantages and challenges in shape transformations by comparing micromachines with the macroscale counterparts and presents the future outlines for the next generation of intelligent shape-morphing micromachines.

## 1. Introduction

Intelligent machines refer to complex mechanical systems that can spontaneously adjust their configurations in response to specific external environments to carry out assigned tasks. Because of excellent environmental sensitivity and adaptability, they have distinctive contributions to many engineering fields including soft robotics [1, 2], wearable devices [3, 4], medical treatments [5, 6], and environment engineering [7]. One of the most significant evaluation criteria for intelligent machines is the shape-morphing capability that would reflect the reconfigurability of the machines in different surroundings and further decide the properties and efficiency of functions. With the rapid development of smart functional materials [8–12], micro/nanofabrication technologies [13–16], small-scale manipulation [17, 18], and actuation strategies [19–22], the shape-morphing performance of intelligent machines has been greatly improved, which leads machines toward miniaturization, intellectualization, and multiple functions. Therefore, intelligent micromachines have been considered with feature sizes from several hundred micrometers down to several micrometers. Intelligent micromachines with programmable shape-morphing properties in response to the change in external stimuli, such as acoustic [23, 24], optic [10, 25, 26], electro [27], thermal [12, 28], magnetic [29, 30], and chemical fields [31], are constantly being explored and created, which have important prospects on small-scale engineering applications, such as biomedicine [29, 32, 33], microfluid channels [34–36], and micromanipulators [37].

Recently, several reviews have made a systematic overview of shape-morphing actuators or robots at microscales. Most of them focus on the progress in advanced materials and fabrications for shape transformations. For example, Hines et al. [38] made a detailed overview of the soft actuators for small-scale robotics classified by different stimulus methods and material types. Spiegel et al. [39] gave a report focused on advanced materials for microscale 4D printing and discussed the critical barriers in the material design and prospective applications. Moreover, Hu et al. [40] opened up another perspective involving soft micro/nanorobotics with flexible components in terms of their architecture designs, fabrication methods, and actuation mechanisms. However, the core issue for the functionalities of intelligent micromachines lies in how to design and realize the expected shape morphing, which is usually affected by various internal and external factors. A review on the shape-morphing characteristic of intelligent micromachines is still absent to date. This review makes a wide-ranging summary of principles and methods for the construction of shape-morphing machines at microscales, including the dimensions, modes, realizations, and applications of shape-morphing intelligent micromachines, and highlights the uniqueness and potential difficulties by comparing them to the macro's ones.

In Section 2, we present the shape-morphing dimensions of intelligent micromachines, classified by the dimensional features before and after deformations. Further, the influences of material selections, design methodologies, and mechanisms of shape-morphing modes are summarized in Section 3. Next, the effective control and actuation strategies for shape morphing of intelligent micromachines are discussed in Section 4. Also, various applications of shape-morphing micromachines concerning their corresponding dimensions, modes, and realization methods are given in Section 5. Besides, the conclusions together with a broader perspective for future researches about shape-morphing micromachines are presented in Section 6.

## 2. Dimensions of Shape Morphing

The shape-morphing performance of intelligent micromachines essentially reflects their spatio-temporal deformation ability. Specifically, the shape-morphing process can be regarded as the fourth dimension related to time alongside the three dimensions of space. Therefore, the shape-morphing dimensions, which contain the space and time dimensions, define the shape-morphing characteristics of micromachines and present a worthwhile issue to be investigated. In general, the shape-morphing dimensions of intelligent micromachines mainly depend on the fabrication methods because the structural configurations and the deformation trends have been almost determined once the structures are formed.

Due to the development of micro/nanofabrication technologies, the explorations to create shape-morphing machines at small scales appear to be flourishing. As the feature size decreases, however, the difficulty of fabrication for shape-morphing microstructures increases significantly. At a smaller scale, higher positioning precision and forming resolution are required during fabrication. In the former, subtle errors in positioning will bring serious influences on the shape-morphing properties of printed structures. The latter determines the smallest feature size of the shape-morphing microunits, which is affected by some practical factors, such as inevitable deformation of materials in the manufacturing process, external temperatures, fabrication speed, and setting time. Furthermore, it is indispensable for achieving shape morphing to require structural regulation during fabrication. For example, in the multimaterial fabrication mode, the regulation is focused on improving the bonding strength and the deformation compatibility of different materials at the interface; in the single-material-single-step mode, the whole rigid structures require the regulation on the clearance fit among different components, while the study on the flexible structures is concentrated on regulating the material distributions to improve the deformation abilities. Therefore, the fabrication of shape-morphing micromachines with spatio-temporal coupling is a subject of multidisciplinary, concerning the micro-electro-mechanical system (MEMS), materials, optics, physics, and chemistry, which affects the dimension transformation performance of shape morphing. Here, we divide the dimension characteristics of shape-morphing micromachines into four categories, i.e., 1D-to-2D, 1D-to-3D, 2D-to-3D, and 3D-to-3D shape morphing.

### 2.1. 1D-to-2D Shape Morphing

**Figure 1:**
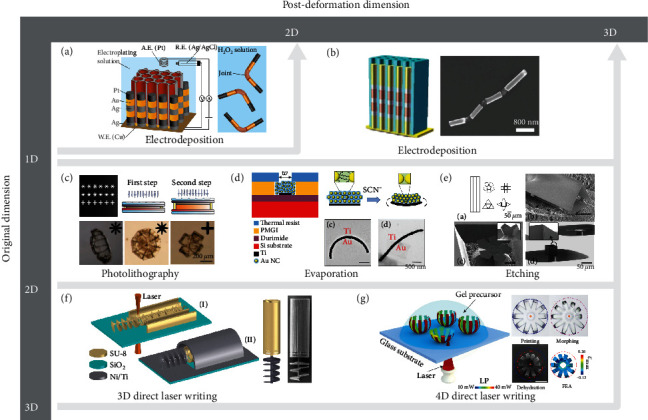
Shape-morphing dimensions of intelligent micromachines: (a) electrodeposition for 1D-to-2D shape morphing [41]. Reproduced with permission. Copyright 2017, American Chemical Society; (b) electrodeposition for 1D-to-3D shape morphing [42]. Reproduced with permission. Copyright 2016, Wiley-VCH; (c) 2D photolithography for 2D-to-3D shape morphing [6]. Reproduced with permission. Copyright 2013, Wiley-VCH; (d) evaporation for 2D-to-3D shape morphing [50]. Reproduced with permission. Copyright 2018, Wiley-VCH; (e) etching for 2D-to-3D shape morphing [51]. Reproduced with permission. Copyright 2010, American Institute of Physics; (f) 3D direct laser writing for 3D-to-3D shape morphing [29]. Reproduced with permission. Copyright 2015, Wiley-VCH; (g) 4D direct laser writing for 3D-to-3D shape morphing [55]. Reproduced with permission. Copyright 2020, American Association for the Advancement of Science.

1D-to-2D shape morphing often happens in large-slenderness-ratio microrods with a flexible joint. The rods experience free 2D folding to angles when actuated by the relative motion of two rigid ends around the joint. The length of the microrods is usually several micrometers while the diameter is generally at the nanoscale. Therefore, the major fabrication technology is electrodeposition for the 1D-to-2D shape morphing, which can produce *in situ* metallic coatings on a conductive material under the action of an electric current. For example, Yoshizumi et al. [41] employed the electrodeposition method to fabricate a 1D microrod with a nanometer-scale diameter as shown in Figure 1(a). In their work, silver, gold, and platinum were electro-posited in turn with the microtube template to form the microrod, and the length was tuned by the electrodeposition duration. Besides, a flexible joint was made of a tubular polymer membrane consisting of bilayered polyelectrolytes, whose folding angle was controlled by external environments.

### 2.2. 1D-to-3D Shape Morphing

Li et al. [42] (Figure 1(b)) and Jang et al. [43] extended the shape morphing dimension of 1D microrods to 3D by increasing the number of flexible joints. They fabricated a fish-like 1D microstructure by using sequential template electrodeposition with different materials. Under the magnetic propulsion, the nickel segment periodically bent the body and caudal fin into a 3D shape and realized oscillating swimming motions. With the same fabrication method, Ahmed et al. [23] and Gao et al. [44] realized 1D-to-3D shape morphing by increasing the length of the flexible part to a considerable value that is comparable in size to the rigid zone. Under the specific external stimuli, the 1D micromachine was triggered to realize 3D wave-like shape morphing on its flexible part.

### 2.3. 2D-to-3D Shape Morphing

2D-to-3D shape morphing is the most common one because it enables various shape-morphing modes using some relatively simple fabrication methods. 2D photolithography is the most popular fabrication technology for manufacturing micromachines with the 2D-to-3D shape-morphing function. Through adjusting the degrees of photo-polymerization reaction on polymer chains, the distribution of crosslinking densities can therefore be designed. Jeon et al. [45], Na et al. [46], and Fusco et al. [6] (Figure 1(c)) printed 2D-to-3D shape-morphing microstructures with the aid of masks. The regions without masks obtained sufficient crosslinking while the ones under the masks received less. In this way, they could realize a programmable photo-polymerization pattern. Martella et al. [47, 48] and Xiong et al. [49] adopted mask-free direct laser polymerization to fabricate microstructures, where the spatial distribution of crosslinking extents might be tuned through controlling the laser powers and the corresponding scan speeds.

Evaporation is another alternative effective fabrication method for 2D-to-3D shape-morphing micromachines. Zhang et al. [50] fabricated heterogeneous microbilayers through sequential Ti evaporation and Au nanocrystal spin-coating. After a ligand exchange process, the microbilayers performed 2D-to-3D bending with an obvious curvature, while the ones without ligand exchange showed only a slight curvature (Figure 1(d)). Moreover, Honschoten et al. [51] employed the plasma etching method to remove redundant regions, and only several narrow segments were left as hinges on the 2D thin film for further programmable 2D-to-3D shape morphing.

### 2.4. 3D-to-3D Shape Morphing

As one of the most challenging tasks, 3D-to-3D shape morphing is highly desirable for sophisticated deformations toward potential engineering requirements. So far, several effective fabrication methods for 3D-to-3D shape morphing have already been proposed due to the recent progress of micro/nanofabrication technologies. Using 3D direct laser writing, Huang et al. [29] (Figure 1(f)), Avci et al. [52], and Vizsnyiczai et al. [53] fabricated complex 3D micromachines with rigid independent components. During the process of direct laser writing, the photo-initiators of photoresist SU-8 absorbed photons and underwent photochemical reactions to form a strong acid, which then catalyzed the crosslinking reaction in photoresist in the process of postdrying and heat preservation. The 3D-to-3D shape morphing was caused by relative motion control among different functional components.

However, the construction of 3D structures at microscale with high shape-morphing freedoms posed challenges because of the lack of applicable materials and effective microfabrication techniques. Jin et al. [54] developed an advanced 4D direct laser writing strategy for reconfigurable compound micromachines as shown in Figure 1(g). The heterogeneous chemical and mechanical properties of stimulus-responsive hydrogels were spatially distributed into high-resolution 3D structures by modulating the femtosecond laser parameters and finally realized 3D-to-3D shape morphing at the microscale.

### 2.5. Section Summaries

With the development of micro/nanofabrications, the configuration transforming of shape-morphing micromachines becomes more sophisticated; namely, the spatial dimension of micromachines before and after shape morphing increases. Pioneering researchers have proposed effective fabrication methods for 3D-to-3D shape morphing, which greatly enriches the structure forms and shape-morphing patterns. However, multimaterial micromachines, which allow the micromachines to have various desired shape-morphing performance, can only be fabricated for low dimensional shape morphing. Therefore, future studies can be focused on exploring the programmable 3D-to-3D shape morphing of multimaterial micromachines.

## 3. Modes of Shape Morphing

Shape-morphing modes describe the reconfiguration performance among different components or regions of intelligent micromachines, which determine the configurations before and after the shape-morphing process and even influence the functions. In general, the shape-morphing mode of the micromachines is related to their material properties, distributions of materials, and relative motions among different components.

In recent studies, almost all the shape morphing of intelligent micromachines exhibits simple and single modes compared to the ones at the macroscale, as it is hard to realize relatively complex shape-morphing modes at such a small scale due to the limitation of fabrication resolution. To ensure the integrity of structures and functions for the intelligent micromachines, simple and single shape-morphing modes have to be selected. Unfortunately, simple shape-morphing modes constrict the microstructures for large and complex deformation and directly affect the functions for further applications.

In this section, the shape-morphing modes employed in intelligent micromachines are discussed. All the modes are divided into three categories about material properties concerning rigid, rigid-flexible mixing, and flexible, which are associated with the number of shape-morphing freedom of the intelligent micromachines.

### 3.1. Shape Morphing of Rigid Structures

For rigid micromachines, shape morphing comes from relative motions between different rigid components, where translating and rotating are two main shape-morphing modes.

#### 3.1.1. Translating

**Figure 2:**
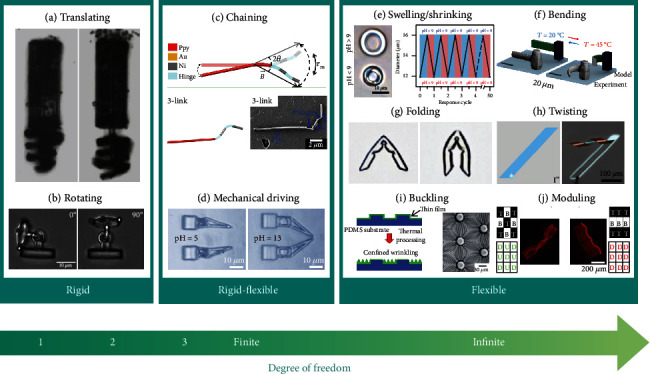
Shape-morphing modes of intelligent micromachines: (a) translating [29]. Reproduced with permission. Copyright 2015, Wiley-VCH; (b) rotating [52]. Reproduced with permission. Copyright 2017, Wiley-VCH; (c) chaining [43]. Reproduced with permission. Copyright 2015, American Chemical Society; (d) mechanical driving [56]. Reproduced with permission. Copyright 2020, Springer Nature; (e) swelling/shrinking [36]. Reproduced with permission. Copyright 2019, The Royal Society of Chemistry; (f) bending [12]. Reproduced with permission. Copyright 2019, Springer Nature; (g) folding [57]. Reproduced with permission. Copyright 2019, Wiley-VCH; (h) twisting [58]. Reproduced with permission. Copyright 2019, Springer Nature; (i) buckling [59]. Reproduced with permission. Copyright 2010, The Royal Society of Chemistry; (j) modular assembling [60]. Reproduced with permission. Copyright 2019, Wiley-VCH.

Huang et al. [29] proposed a rigid micromachine consisting of a cylinder and a magnetic screw fabricated by using SU-8 photoresist which was locally deposited with Ni/Ti nanometer-thin layers. Under effective magnetic manipulations, the magnetic screw was actuated by the external magnetic field and obtained lift to translate along to the cylinder (Figure 2(a)). Since all the components were rigid without local deformation, the coordinate of an arbitrary point *i* on the mobile component *m*_*i*_ after the rigid translating along vector (*t*_*x*_, *t*_*y*_, *t*_*z*_) can be described as

(1) $$\begin{array}{c} m_{i}=TM_{i}, \end{array}$$

where *M*_*i*_ is the initial coordinate and **T** is the translating transformation, which can be expressed as [61].

(2) $$\begin{array}{c} T\left(t_{x},t_{y},t_{z}\right)=\left[\begin{array}{cccc} 1 & 0 & 0 & t_{x}\\ 0 & 1 & 0 & t_{y}\\ 0 & 0 & 1 & t_{z}\\ 0 & 0 & 0 & 1 \end{array}\right]. \end{array}$$

Employing Equations (1) and (2), one can calculate the arbitrary-point coordinate on the mobile component and construct the configuration of micromachines after shape morphing.

#### 3.1.2. Rotating

Avci et al. [52] designed an articulated micromachine with a mobile head and a base. They realized a controllable out-of-plane rotational motion of the head under an optical manipulation (Figure 2(b)) through applying suitable power of optical tweezers. The coordinate of an arbitrary point *i* on the relative rotating component after relative rotating along an arbitrary axis $\vec{A}\left(A_{x},A_{y},A_{z}\right)$ can be obtained by

(3) $$\begin{array}{c} m_{i}=R_{A}M_{i}, \end{array}$$

where the rotating transformation **R**_**A**_ is [61].

(4) $$\begin{array}{c} R_{A}\left(\theta \right)=\left[\begin{array}{ccc} c+\left(1-c\right)A_{x}^{2} & \left(1-c\right)A_{x}A_{y}-{sA}_{z} & \left(1-c\right)A_{x}A_{z}+{sA}_{y}\\ \left(1-c\right)A_{x}A_{y}+{sA}_{z} & c+\left(1-c\right)A_{y}^{2} & \left(1-c\right)A_{y}A_{z}-{sA}_{x}\\ \left(1-c\right)A_{x}A_{z}-{sA}_{y} & \left(1-c\right)A_{y}A_{z}+{sA}_{x} & c+\left(1-c\right)A_{z}^{2} \end{array}\right], \end{array}$$

with *θ* the rotating angle around $\vec{A}$, *c* = cos*θ*, and *s* = sin*θ*.

#### 3.1.3. Combination of Translating and Rotating

Moreover, the combination of translating and rotating was employed to increase the total degrees of shape-morphing freedom of rigid micromachines. Vizsnyiczai et al. [53] proposed a 3D interconnected micromotor including a ramp, an axis, and a rotor. Through the propulsion from bacteria, the rotor was triggered to achieve a rotating motion around a fixed axis and a translating motion along the axis. Similar to the foregoing analyses, the coordinate of an arbitrary point *i* on the relative shape-morphing component after the shape morphing of rigid structures can be deduced by

(5) $$\begin{array}{c} m_{i}=MM_{i}, \end{array}$$

where **M** is the transformation matrix of this shape morphing, which is the product of the translating transformation and the rotating transformation, i.e.,

(6) $$\begin{array}{c} M=T\left(t_{x},t_{y},t_{z}\right)R_{A}\left(\theta \right). \end{array}$$

### 3.2. Shape Morphing of Rigid-Flexible Hybrid Structures

#### 3.2.1. Chaining

Concerning more degrees of freedom, a chain-like shape-morphing mode was proposed with one or several flexible joints embedded into the rigid rods [41–43]. Each flexible joint can provide two spatial rotational degrees of freedom due to its soft-material properties (Figure 2(c)). Therefore, the total degrees of freedom grow in multiples of two as the number of joints increases. Moreover, inspired by the microorganisms, such as Escherichia coli consisting of a rigid head and a flexible long tail, Ahmed et al. [23] and Gao et al. [44] demonstrated an advanced chaining shape-morphing mode with rigid-flexible mixing structures for micromachines. The flexible sections experienced reconfiguration from a line to a rotating spiral during the swimming process. In this case, one can capture the end-point coordinate of the *j*-th chaining rods after shape-morphing (with the start-point of the first rod as the origin) by

(7) $$\begin{array}{c} \vec{O_{j}}=\left[\begin{array}{c} \overset{j}{\underset{i=1}{\sum }}r_{i}\sin\theta_{i}\cos\varphi_{i}\\ \overset{j}{\underset{i=1}{\sum }}r_{i}\sin\theta_{i}\sin\varphi_{i}\\ \overset{j}{\underset{i=1}{\sum }}r_{i}\cos\theta_{i} \end{array}\right], \end{array}$$

where *j* ∈ [1, *n*], *n* is the number of chains. *r*_*i*_(*i* = 1, 2, ⋯, *n*), *θ*_*i*_, and *φ*_*i*_ are the length, the zenith angle with *z*-axis, and the azimuth angle with *x*-axis projecting in the *x*O*y* plane of the *i*‐th chain, respectively. According to Equation (7), a chaining rod with better shape-morphing ability can be achieved by increasing the number of flexible joints or expanding the angle ranges. When *n* → +∞ within a fixed-length rod, the chaining rod turns to a completely flexible rod with infinite degrees of freedom.

#### 3.2.2. Mechanical Driving

Another shape-morphing mode of rigid-flexible hybrid structures is the mechanical driving. Ma et al. [56] developed a programmable artificial musculoskeletal system by combing a stiff SU-8 “skeleton” and a pH-responsive smart “muscle” into 3D micromachines and demonstrated that the mechanical “skeleton” could be driven by the “muscle” under specific pH stimuli (Figure 2(d)). The shape-morphing analysis of the mechanical driving is similar to the rigid-structure cases.

### 3.3. Shape Morphing of Flexible Structures

Flexible materials provide infinite degrees of freedom for shape morphing and construct multiple shape-morphing modes, which have become a research hotspot over the past decade.

#### 3.3.1. Swelling/Shrinking

Swelling/shrinking is the simplest flexible shape morphing, which refers to a structure with homogeneous material. The final configuration of the structure is the similarity transformation of the initial one. Therefore, when the flexible structure undergoes homogeneous swelling or shrinking from the reference state *x*_*i*_ to the current state *X*_*K*_, its deformation gradient **S**, which is defined as the partial derivative of *x*_*i*_ concerning *X*_*K*_ [62], i.e.,

(8) $$\begin{array}{c} S=\frac{\partial x_{i}\left(X,t\right)}{\partial X_{K}}=\left[\begin{array}{ccc} s & 0 & 0\\ 0 & s & 0\\ 0 & 0 & s \end{array}\right], \end{array}$$

where *s* is the swelling/shrinking ratio. Equation (8) constructs a corresponding relationship between the reference configuration and the current one, and thus, the shape morphing configuration can be predicted when *s* is given. Hu et al. [36] fabricated a pH-sensitive cylindrical microfluidic machine as shown in Figure 2(e), in which the structure could be precisely controlled for uniform swelling or shrinking. Another pH-dependent swelling micromachine was demonstrated by Lee et al. [63], whose swelling performance was tuned by the layer distance in the layer-by-layer lithography. Zeng et al. [64] printed a light-fueled cubic-shape micromachine, which had ~20% contraction when it was triggered by a focused laser beam.

#### 3.3.2. Bending

Bending is a global shape morphing with a smooth curvature over the structure. The bending happens when there exists a strain mismatch along the thickness direction. Zhang et al. [50], Xiong et al. [49], and Hippler et al. [12] (Figure 2(f)) fabricated bilayer microbeams with large aspect ratios. The two layers were under different degrees of deformation, leading to the bending along the length. However, the situations become distinct when the width is comparable to the length. Stoychev et al. [65] investigated the bending properties of rectangular bilayered thin films with different aspect ratios and relative thicknesses. It showed several bending directions, i.e., long-side, all-side, diagonal, and mixing, which were dominated by the ratio of length to width. Relative energetic analysis, finite-element modeling, and microscopy snapshots with various bending directions were exhibited in their work. Moreover, by programming the size and distribution of laser writing voxels or unilateral ultraviolet exposure, Zhang et al. [66] and Jamal et al. [35] reported a bending mode with stress gradients along the thickness direction. Further, various compound bending modes were proposed by embedding bending units into local regions of micromachines for specific shape-morphing requirements [47, 54, 63, 67].

It is well-known that the curvature is the core parameter for bending, which can be obtained by the classical theory of Timoshenko's beam [68] based on the physical balance of structures and geometric coordination at the bilayer interface. For instance, the curvature *κ* of the *n*-layer structure can be solved as [67, 69, 70].

(9) $$\begin{array}{c} \kappa =\frac{6\sum_{i=1}^{n}\left(2E_{i}a_{i}\left(\sum_{j=1}^{i-1}a_{j}+\left(a_{i}/2\right)\right)\sum_{k=1}^{n}\left(E_{k}a_{k}\left(\sum_{l=1}^{i-1}\left(\varepsilon_{l+1}-\varepsilon_{l}\right)-\sum_{l=1}^{k-1}\left(\varepsilon_{l+1}-\varepsilon_{l}\right)\right)\right)\right)}{\sum_{i=1}^{n}\left(E_{i}a_{i}\left(\sum_{j=1}^{n}E_{j}{a_{j}}^{3}+6\left(\sum_{j=1}^{i-1}a_{j}+\left(a_{i}/2\right)\right)\sum_{k=1}^{n}\left(E_{k}a_{k}\left(a_{i}-a_{k}+2\left(\sum_{l=1}^{i-1}a_{l}-\sum_{l=1}^{k-1}a_{l}\right)\right)\right)\right)\right)}, \end{array}$$

where *E*_*i*_, *a*_*i*_, and *ε*_*i*_ represent the Young's modulus, thickness, and strain of the *i*‐th layer, respectively. For the case of bilayer beams (*n* = 2), the expression of curvature can be simplified as

(10) $$\begin{array}{c} \kappa =\frac{1}{a_{1}+a_{2}}\cdot \frac{6\left(\varepsilon_{2}-\varepsilon_{1}\right){\left(1+p\right)}^{2}}{3{\left(1+p\right)}^{2}+\left(1+pq\right)\left(p^{2}+\left(1/\left(pq\right)\right)\right)}, \end{array}$$

where *p* = *a*_1_/*a*_2_, *q* = *E*_1_/*E*_2_. It can be found from the above two equations that the bending ability of microbeam can be improved by increasing the difference between the strains of two adjacent layers, decreasing the total thickness, or choosing suitable thickness ratios or Young's modulus ratios, etc.

#### 3.3.3. Folding

Folding is the localized bending in narrow deformable regions of structures, which essentially behaves as hinges. Folding refers to a relative rotating around the hinge and finally exhibits an angular shape. Honschoten et al. [51] fabricated a microstructure with a silicon nitride layer and then created hinges by photolithography and plasma etching of the layer to a thinner thickness as the crease for folding. Chen et al. [57] and Jin et al. [54] embedded narrow bilayer structures into micromachines for obviously fixed-point folding (Figure 2(g)). In general, the folding direction is predesigned, rather than random swings like chaining. The folding angle *θ* is the key parameter for folding, which is related to the curvature *κ* of the bending part. *θ* and *κ* satisfy the following geometric relation [71].

(11) $$\begin{array}{c} \theta =\pi -\kappa L, \end{array}$$

where *L* is the hinge length. Therefore, the folding ability changes with the bending deformation of the hinge.

#### 3.3.4. Twisting

Twisting is another flexible shape-morphing mode, which can be viewed as generalized bending. However, the bending direction angle along with the long axis of twisting is adjustable, and it finally exhibits a helix shape. Xu et al. [58] proposed a microdroplet-guided shape-morphing strategy of microstructures for programmable twisting (Figure 2(h)). The twisting direction can be precisely controlled by selecting a trigger point of microdroplets. Mourran et al. [72] and Jeon et al. [45] fabricated twisting shape-morphing helixes by embedding specific-angle mismatches between the principal axis of stress and the long axis of stripes.

The twisting configuration is characterized by the curvature *κ* and the pitch *p* [45, 73, 74], which are determined by the thickness *h*, the width *w*, the angle between the twisting direction, and the long axis *θ*. The design criterion and improving strategy of twisting are similar to bending because it is a generalized bending. When *θ* = 0° or 90°, the twisting shape morphing degenerates into bending. For further quantitative analysis, two dimensionless parameters were employed by Jeon et al. [45] in a shape-morphing phase diagram, i.e., the dimensionless helix pitch $\tilde{p}=\kappa p$ and the dimensionless width $\tilde{w}=w\sqrt{\kappa /h}$, which can systematically capture the twisting rules for further tuning and optimizing.

#### 3.3.5. Buckling

Buckling of structures with periodic and regular array patterns appears when the top and bottom surfaces of a membrane structure experience differential deformations. Wang et al. [75] and Hendricks et al. [59] (Figure 2(i)) realized programmable buckling in thin films when they exerted compressive or tensile deformations on substrates. The related physical mechanism, control, and prevention strategies were also proposed in their investigations. Buckling of the film happens when the strain reaches the following critical strain *ε*_*c*_ [76, 77].

(12) $$\begin{array}{c} \varepsilon_{c}=\frac{1}{4}{\left(\frac{3{\bar{E}}_{\text{s}}}{{\bar{E}}_{\text{f}}}\right)}^{2/3}, \end{array}$$

where $\bar{E}=E/\left(1-\nu^{2}\right)$, *E* and *ν* are Young's modulus and Poisson's ratio, respectively. The subscripts *f* and *s* represent the film and the substrate, respectively. Therefore, it is easier to realize buckling by decreasing the ratio of ${\bar{E}}_{s}$ to ${\bar{E}}_{f}$, leading to a smaller *ε*_*c*_. Moreover, the wavelength *λ* and the amplitude *A* of the buckling pattern can be quantified as [59, 76, 77]

(13) $$\begin{array}{c} \lambda =2\pi d{\left(\frac{{\bar{E}}_{\text{f}}}{3{\bar{E}}_{\text{s}}}\right)}^{1/3}, \end{array}$$

(14) $$\begin{array}{c} A=d{\left(\frac{\varepsilon }{\varepsilon_{\text{c}}}-1\right)}^{1/2}, \end{array}$$

where *ε* and *d* are the applied strain and the thickness of the membrane, respectively. And thus, the buckling configuration can be predicted and further tuned.

#### 3.3.6. Modular Assembling

When the micromachine is assembled by a series of identical shape-morphing modules, it can produce modulated structural transformations. The modular structures have distinctive advantages to achieve complex and programmable deformation, which is an alternative strategy to break the limitation of single and simple deformation of the current intelligent micromachines. Zhou et al. [60] explored the pattern buckling modular assembling with 2 × 3 and 3 × 3 arrays (Figure 2(j)). They furtherly found that the buckling direction failed to adopt the desired shapes when there exhibited opposite-direction modules inserted into the center of the row. Jamal et al. [35] fabricated 3D microstructures with interconnected modular shape-morphing segments. This periodic pattern had versatility in self-assembling geometries. Cui et al. [30] also introduced the modular assembling strategy in their structural design of shape-morphing micromachines. They demonstrated assemble modular units for encoded morphing information into the letter of the alphabet and finally obtained a bird-shaped micromachine with complex and various motions. Huang et al. [55] extended the dimension of modular units into 3D, proposing a programmable modular design for constructing sophisticated 3D-to-3D shape-morphing microstructures by assembling 4D microbuilding blocks. A microscale transformer was fabricated and devised, capable of changing from a racecar to a humanoid robot. Qu et al. [78] fabricated 3D microlattices with shape-morphing modular units and obtained 3D metamaterials with tunable thermal-expansion coefficients.

In practice, the well-established Denavit-Hartenbetg parameters are utilized for structural design and kinematic analysis of modular assembling [55]. The relative movement allowed at the junction is quantified by a transformation [*Z*_*i*_], whereas the dimensions of each link are defined by a separate transformation [*X*_*i*_]. In this context, the transformation of the end of *n* building blocks is then described as

(15) $$\begin{array}{c} \left[T\right]=\left[Z_{1}\right]\left[X_{1}\right]\left[Z_{2}\right]\left[X_{2}\right]\cdots \left[Z_{n-1}\right]\left[X_{n-1}\right], \end{array}$$

where

(16) $$\begin{array}{c} \left[Z_{i}\right]=\text{Tran}{\text{s}}_{z_{i}}\left(d_{i}\right){\text{Rot}}_{z_{i}}\left(\theta_{z_{i}}\right), \end{array}$$

(17) $$\begin{array}{c} \left[X_{i}\right]=\text{Tran}{\text{s}}_{x_{i}}\left(R_{i,i+1}\right){\text{Rot}}_{x_{i}}\left(\alpha_{i,i+1}\right), \end{array}$$

*d*_*i*_, *θ*_*z*_*i*__, *R*_*i*,*i*+1_, and *α*_*i*,*i*+1_ are the DH parameters, which may be implemented into the modular assembling system for kinematic shape-morphing predictions and optimizations. Likewise, Huang et al. [55] demonstrated that the DH matrix can be employed to facilitate the inverse and forward design of complex and arbitrary modular assembling cases.

### 3.4. Section Summaries

Intelligent micromachines have provided unique and various shape-morphing modes no matter in rigid, rigid-flexible, or flexible materials. However, they still have their challenges in further micromachine design. Shape-morphing modes of rigid structures, i.e., translating and rotating or their mixing, have advantages in precise relative motion control among components because of their limited shape-morphing degrees of freedom. Nevertheless, the limitations of the freedom degree hinder the development towards multiple and sophisticated functions. Moreover, rigid microstructures have poor environmental and mechanical compliance, which is not suitable for applications in biomedical engineering. For rigid-flexible chaining micromachines, the embedded flexible joints bring real-time dynamic configurations transforming following the external environment. It greatly improves environmental compliance and adaptability. However, the random shape-morphing property increases the difficulty in control. The shape-morphing analyses on the flexible structures encounter many bottlenecks in recent years because of the infinite degrees of freedom and nonlinear deformation properties, such as the constructions of soft-material constitutive models, the analysis of interface mismatching, and the influence of size effects.

## 4. Realizations of Shape Morphing

Another important factor worth considering is the realization method of shape morphing, which denotes the actuation and control strategies to exhibit specific deformation and further locomotion of intelligent micromachines. In comparison with shape-morphing modes discussed in Section 3, which summarizes the initial and final configurations design according to the material properties, this section is focused on the dynamic process of shape morphing.

It is generally known that the intelligent machines in the macroscale have various actuation and control methods for shape morphing, which could be classified into four types according to the energy transformation modes: (1) hydraulic drive, which can realize high accuracy control using the incompressibility of liquid; (2) pneumatic drive, which has large actuation speed because of the small viscosity of compressed air and large flow rate; (3) motor drive, a current mainstream driver with convenient energy transfer and quick signal transformation; and (4) advanced drive, utilizing magnetostrictive effect, piezoelectric effect, or electrostatic drive of materials and some smart materials, like shape memory alloy and ultrasonic-/optical-/magnetic-response materials. The function conditions and requirements of machines should be comprehensively considered in the process of selecting the specific actuation and control modes. However, as the size decreases to the micrometer scale, alternative actuation and control modes become much rarer. Because it is difficult to construct and assemble complex and complete control systems in micromachines. Therefore, it becomes feasible to utilize the advanced driven strategy to realize shape-morphing control and untethered actuation. Nevertheless, it is difficult to achieve independent deformation control in different microregions, because most of the stimuli-response microstructures undergo shape morphing under uniform external fields. Therefore, it is still a great challenge to acquire accurate and independent shape-morphing control on different compounds of intelligent micromachines, further constricting programmable locomotion and complex functions. Moreover, the response speeds for the material deformation, and the component movement is of positive correlation with the feature size of the structures. The shape-morphing response becomes faster on intelligent machines as the size decreases, which increases the difficulty in the effective control of the shape-morphing process. It is known from the law of momentum conservation that breaking symmetry and then creating asymmetry is an essential method to realize functional shape morphing of intelligent micromachines. Here, we divide the asymmetric factors into two categories, i.e., external asymmetry and internal asymmetry.

### 4.1. External Asymmetry

#### 4.1.1. Asymmetric Stimulus

**Figure 3:**
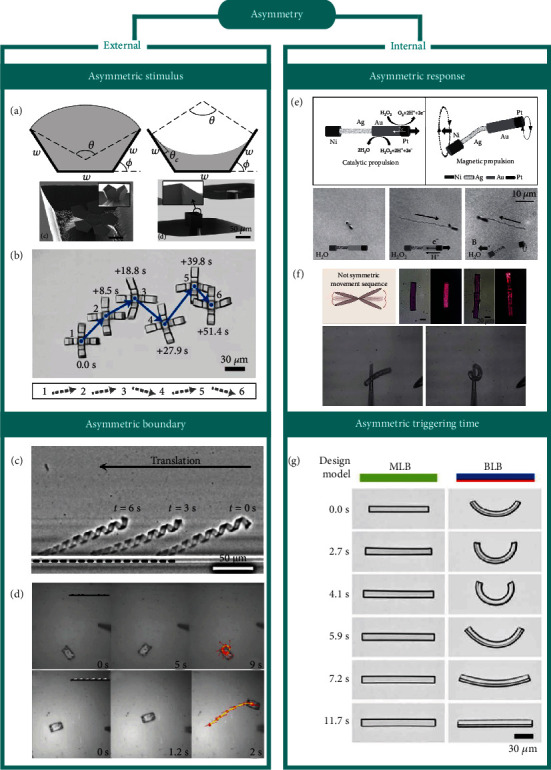
Shape-morphing realizations of intelligent micromachines: (a) [51] and (b) [57] asymmetric stimulus. Reproduced with permissions. Copyright 2010, American Institute of Physics and Copyright 2020, Wiley-VCH, respectively; (c) [72] and (d) [64] asymmetric boundary. Reproduced with permissions. Copyright 2016, Wiley-VCH and Copyright 2015, Wiley-VCH, respectively; (e) [44] and (f) [48] using asymmetric response. Reproduced with permissions. Copyright 2011, Wiley-VCH and Copyright 2017, The Royal Society of Chemistry, respectively; (g) asymmetric triggering time [57]. Reproduced with permission. Copyright 2020, Wiley-VCH.

The external asymmetry involves the external factors responsible for shape morphing, the most important of which is the asymmetric stimulus. It creates spatial variability of the ambient environment for the intelligent micromachines, which could trigger orientable or sequential shape morphing of structures. Since the surface tension dominates rather than the bulk force at the small scale, capillarity becomes an effective mechanism to trigger shape morphing [58, 79–81]. Utilizing the capillary forces, Honschoten et al. [51] developed an origami-based technique for planer microstructures folding into 3D objects (Figure 3(a)). After droplets were dropped on one side of the structures, asymmetric surface tension promoted folding due to the spontaneous evaporation of water. The corresponding theoretical model was put forward to describe the elasto-capillary interaction of the shape-morphing process and the folding tuning method. On the other hand, diffusion emerges as the spatial-temporal variability of solvents in solution. Though it is a random and unpredictable process in a static fluid, the diffusion path could be artificially controlled when the velocity of the fluid is greater than the diffusion speed. Chen et al. [57] proposed a shape-morphing realization strategy to trigger a series of microcrawlers walking along a predesigned diffusion path (Figure 3(b)). Through controlling of injecting directions and rates of the alkaline solution into the acidic solution, the trajectories of neutralization reaction could be manipulated. Subsequently, the microjoints of the microcrawlers deformed in sequence and brought the asymmetric dynamic frictions between feet and substrates, which thus realized directional locomotion. Mourran et al. [72] proposed a similar diffusion-path-control strategy but focused on temperature diffusion to realize the dynamic asymmetry in the shape morphing.

#### 4.1.2. Asymmetric Boundary

The boundary asymmetry is another external asymmetric factor. If we define the asymmetric stimuli discussed in Section 4.1.1 as the dynamic external factor, the asymmetric boundary could be regarded as the static one. Although boundaries constrain the deformation and locomotion of intelligent micromachines near the wall, it could also be employed to create asymmetric situations for the realization of shape morphing.

In nature, some microorganisms control their swimming directions by utilizing the constraints from fixing flagella. Inspired by nature, Mourran et al. [72] achieved a linear translocation of the oscillating microhelices instead of a rotational motion when they approached a wall as shown in Figure 3(c). Meanwhile, the microhelices could obtain a locomotion speed comparable to some helical bacteria. Zeng et al. [64] drove a microwalker on the substrates with predesigned patterns. Owing to the shear off anisotropy, the walker experienced shape-morphing locomotion with preferred directions and final exhibited various motion modes, including random walking, directional walking, rotating, and even jumping (Figure 3(d)).

### 4.2. Internal Asymmetry

The internal asymmetric factors come from the intrinsic attributes of intelligent micromachines, such as the material properties and structure configurations, which are also influenced by the fabrication techniques.

#### 4.2.1. Asymmetric Response

When different regions of intelligent micromachines are triggered by different deformation degrees or stimuli, the whole micromachines realize asymmetric-response shape morphing [43, 47–50, 54, 57, 69]. For example, Gao et al. [44] reported a synthetic hybrid wire-shaped motor at the microscale. It consisted of a flexible, a catalytic-response, and a magnetic-response segment. When it was immersed in the hydrogen peroxide solution, the catalytic segment triggered the decomposition of hydrogen peroxide, and simultaneously, the magnetic-response segment was driven under the rotating magnetic field, leading to a wire-to-helix reconfiguration and breaking the symmetry of structures for further movements (Figure 3(e)). Different from the multimaterial or multistimuli strategy for an asymmetric response, Martella et al. [48] focused on the use of light to activate liquid crystalline elastomers for nonreciprocal motion. Double stripes with different mixture properties were employed for intelligent micromachines as shown in Figure 3(f), which provided different degrees of shape morphing and finally realized nonsymmetric rapid movement driven by the light within the time scale of seconds.

#### 4.2.2. Asymmetric Triggering Time

Asymmetric triggering time is an effective internal asymmetric factor for the realization of shape morphing. Chen et al. [57] found a sequential deformation effect of pH-response bilayer beams at the microscale. The layer with dense crosslinking density was triggered ahead of the loose one in response to the change in the ambient solution from acidic to alkaline and induced sequential swelling between two layers of the microbeam. This asymmetric triggered effect brought multimode shape morphing to the micromachines and was finally used in the morphing-driven locomotion of microcrawlers (Figure 3(g)).

### 4.3. Section Summaries

Micromachines are still posing big challenges in sophisticated and sequential shape-morphing actuation and control of different functional regions due to the small size. The use of asymmetry provides a promising way to realize shape-morphing micromachines. In future research, new asymmetric mechanisms of shape morphing should be explored and applied to intelligent micromachines. Moreover, hybrid asymmetric factors should be combined for more diverse shape morphing.

## 5. Applications of Shape-Morphing Micromachines

The shape-morphing performance offers the ability of multiple reconfigurations for intelligent micromachines and contributes to various applications in the engineering fields. In comparison with the macroscale shape-morphing machines capable of realizing complex shape-morphing configurations via precise programming for intriguing applications [22, 82–84], the shape-morphing intelligent micromachines can only produce relatively simple shape-morphing patterns due to the limitations of fabrication technologies and control strategies. But intelligent micromachines have advantages in fast shape-morphing response, low actuation strength, and convenient in-mass control due to their small volumes, high flexibility, and strong adaptability. Therefore, they have unique potential applications especially in small-scale or narrow spaces like microfluidic channels [35, 36], biochips [85], and blood vessels [54]. For example, Huang et al. [29] proposed an intelligent micromachine capable of actively carrying out tasks of targeted and triggering delivery of particles, biological materials, and even smaller micromachines under shape morphing driven by magnetic fields. This microtransporter can be used to access remote places of the body for medical treatment. Hu et al. [36] reported a shape-morphing microfluidic machine by integrating a pH-sensitive microring array into a microchannel. The machine could trap particles of different sizes in a multifiltering way. These microrings have potential applications in microobject manipulation and single-cell biology analysis.

However, there still faces many inevitable challenges as the feature size decreases to microscale, including the materials selections, fabrication technologies, structural design, actuation method, and control strategies. The research on the shape-morphing micromachines is still in its infant stage as it involves an emerging and complex interdisciplinary subject. Most current shape-morphing micromachines could only perform fundamental shape morphing to achieve single and simple functions.

**Figure 4:**
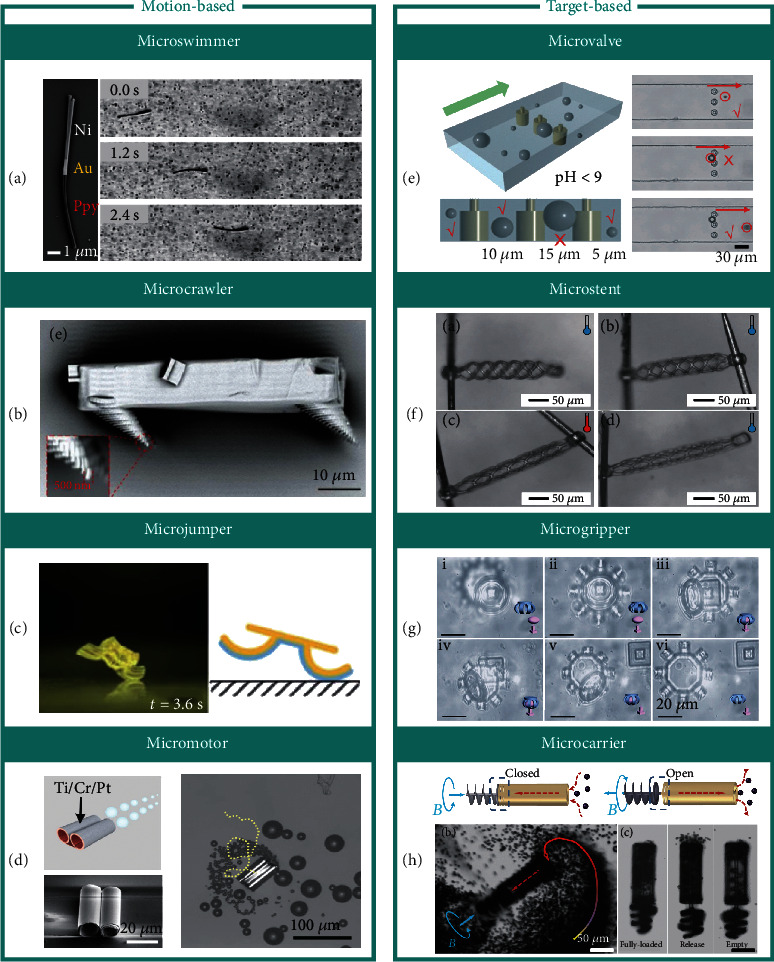
Applications of shape-morphing micromachines. (a–d) Motion-based micromachine. (a) Microswimmer [23]. Reproduced with permission. Copyright 2016, American Chemical Society; (b) Microcrawler [64]. Reproduced with permission. Copyright 2015, Wiley-VCH; (c) Microjumper [86]. Reproduced with permission. Copyright 2010, The Royal Society of Chemistry; (d) Micromotor [58]. Reproduced with permission. Copyright 2019, Springer Nature. (e–h) Target-based micromachine. (e) Microvalve [36]. Reproduced with permission. Copyright 2019, The Royal Society of Chemistry; (f) Microstent [88]. Reproduced with permission. Copyright 2019, Wiley-VCH; (g) Microgripper [66]. Reproduced with permission. Copyright 2019, American Chemical Society; (h) Microcarrier [29]. Reproduced with permission. Copyright 2015, Wiley-VCH.

Here, we list eight types of applications of intelligent micromachines which are divided into motion- and target-based micromachines, as shown in Figure 4. The motion-based micromachines include microswimmers for swimming in the liquid [23, 30, 41–44], microcrawlers for substrate locomotion [57, 64], microjumpers for jumping [64, 86], and micromotors for marching [52, 53, 58, 87], while the target-based micromachines include microvalves for valve control of microchannels [35, 36], microstents for extending vessels [54, 88], microgrippers for drugs catching [26, 47, 49, 56, 66], and microcarriers for drug carrying and delivery [29, 89, 90]. Each application is summarized and marked with its common strategies according to the shape-morphing dimension, shape-morphing modes, and realization methods discussed above, together with their advantages and limitations (see Table 1 for details).

## 6. Summaries and Perspectives

In nature, shape morphing is an important factor to figure out the life and the inanimate and is endowed on the intelligent micromachines. In this review, we briefly summarize the recent progress on the shape morphing of micromachines from four perspectives: (1) the dimension of shape morphing, which emphasizes the dimension transformation before and after shape morphing; (2) the modes of shape-morphing, which is concentrated on the styles of shape morphing related to the material properties and the deformation freedom; (3) the realization methods of shape morphing, which describes the strategies for actuation and control by creating asymmetry on micromachines; and (4) the applications of shape morphing, in which the common applications of shape-morphing micromachines are discussed. All these perspectives point out the significance and indispensability of shape-morphing abilities in intelligent micromachine toward engineering applications, such as targeted drug delivery and release [91, 92], disease diagnosis and treatment [93], environmental monitoring [94], and intelligent microfluidic chips [34]. Although great progress has been achieved in the shape-morphing micromachines, there inevitably exist certain difficulties. For example, it still requires higher fabrication precision and stronger structure forming regulation for high-dimension shape morphing. Moreover, the current shape-morphing modes are quite simple and single. Micromachines with large and complex shape morphing are still absent. Besides, the realization methods of shape morphing are rare at the microscale compared to the macroscales, which increases the difficulty in accurate and programmable control.

As the development trend of the current technologies is moving to high precision, high efficiency, miniaturization, and intellectualization, intelligent micromachines, especially those with the shape-morphing performance, have already become a focus on small-scale research. Predictably, with the development of 4D microprinting technology, the reconfiguration dimension of shape-morphing micromachines will be concentrated on 3D to 3D, which facilitates complex and diverse structure assembling and shape morphing. Modular design strategies may be a new direction towards achieving large, complex, and programmable shape morphing in the future. Furthermore, more asymmetric factors, including internal factors from the materials or external ones from the environments, should be explored to extend the realization approaches of shape morphing. With an effective combination of several asymmetric factors, the intelligent micromachines are on track to achieve precise and alternative shape-morphing control of different regions or further to realize independent parallel control of a single micromachine and swarm collaboration of several micromachines. Inspired by current-related research, one should take into account more applications concerning shape-morphing micromachines, such as location tracking by visual monitoring [95, 96], multiple interaction or cooperation between individuals [97–99], and information perception, data storage/processing, and feedback [100]. The shape-morphing analysis provides a brand-new view to investigate intelligent micromachines. Despite it has many difficulties and limitations at the microscale, the prospects of intelligent shape-morphing micromachines are promising.

## Figures and Tables

**Table 1:** A summary of the shape-morphing characteristics of micromachines with different applications.

	Dimension of shape morphing	Modes of shape morphing	Realizations of shape morphing	Advantages	Limitations	Refs.
Motion-based micromachines						

Microswimmer	(i) 1D →2D (ii) 1D →3D	(i) Chaining (ii) Modular assembling	(i) Asymmetric stimulus (ii) Asymmetric response	(i) Small size (ii) High propulsive forces (iii) Good fluid adaptability	(i) Single swimming posture	[23, 30, 41–44]
Microcrawler	(i) 2D →3D (ii) 3D →3D	(i) Swelling/shrinking (ii) Folding (iii) Bending	(i) Asymmetric stimulus (ii) Asymmetric boundary (iii) Asymmetric response (iv) Asymmetric triggering time	(i) Fast locomotion speed (ii) Controllable trajectory (iii) Good terrain adaptability	(i) Single and simple gait	[57, 64]
Microjumper	(i) 3D →3D	(i) Swelling/shrinking (ii) Buckling	(i) Asymmetric stimulus (ii) Asymmetric boundary (iii) Asymmetric response	(i) Fast snap-through speed (ii) High power density	(i) Hard to repeat continuously	[64, 86]
Micromotor	(i) 2D →3D (ii) 3D →3D	(i) Translating (ii) Rotating (iii) Bending	(i) Asymmetric stimulus (ii) Asymmetric response	(i) Fast actuation speed (ii) Tunable moving direction	(i) Need specific actuation fuel	[52, 53, 58, 87]

Target-based micromachines						

Microstent	(i) 3D →3D	(i) Swelling/shrinking (ii) Folding (iii) Bending	(i) Asymmetric response	(i) Large extending degree (ii) Fast response speed	(i) Weak bearing capacity	[54, 88]
Microvalve	(i) 2D →3D (ii) 3D →3D	(i) Swelling/shrinking (ii) Modular assembling	(i) Asymmetric response	(i) Fast response speed (ii) Reversible switching (iii) Precise particle selection	(i) Only for regular-shaped particles	[35, 36]
Microgripper	(i) 2D →3D (ii) 3D →3D	(i) Bending (ii) Mechanical driving	(i) Asymmetric response	(i) Precise object grasping (ii) Site-specific releasing	(i) Undesired adhesion between the gripper and objects	[26, 47, 49, 56, 66]
Microcarrier	(i) 3D →3D	(i) Translating (ii) Rotating (iii) Bending	(i) Asymmetric stimulus (ii) Asymmetric response	(i) Controllable particles collecting and releasing (ii) Narrow gap transporting	(i) Complex control strategy	[29, 89, 90]
